# ‘Teleonomes in Tatters’: The Welfare Costs of Selecting Dogs for Extreme Phenotypes, and the Case for Change

**DOI:** 10.3390/ani16182867

**Published:** 2026-09-11

**Authors:** Paul D. McGreevy, David J. Mellor, Dan O’Neill, Rowena M. Packer, Cathrynne Henshall, Cristina L. Wilkins

**Affiliations:** 1 Faculty of Science, Sydney School of Veterinary Science, University of Sydney, Sydney, NSW 2006, Australia; 2 Animal Welfare Science and Bioethics Centre, School of Veterinary Science, Massey University, Palmerston North 4442, New Zealand; d.j.mellor@massey.ac.nz; 3 Veterinary Epidemiology, Economics and Public Health, The Royal Veterinary College, Hawkshead Lane, North Mymms, Hatfield AL9 7TA, Herts, UK; doneill@rvc.ac.uk; 4 Department of Clinical Science and Services, The Royal Veterinary College, Hawkshead Lane, North Mymms, Hatfield AL9 7TA, Herts, UK; rpacker@rvc.ac.uk; 5 School Agricultural, Environmental and Veterinary Sciences, Southern Cross University, Lismore, NSW 2678, Australia; info@horselogic.com.au; 6 School of Environmental and Rural Sciences, University of New England, Armidale, NSW 2351, Australia; cwilki23@myune.edu.au

**Keywords:** extreme morphotypes, Five Domains Model, allostatic load, fitness for purpose, canine welfare, innate health assessment, brachycephalic

## Abstract

The domestic dog is the most morphologically diverse domesticated mammalian species. That diversity has arisen from deliberate, sustained artificial selection over the most recent 1% of the domestic dog’s evolutionary history. However, artificial selection has pushed many individual breeds so far from the ancestral canine form that many dogs within these breeds can no longer perform the critical biological functions their bodies evolved to serve. Indeed, much of the popularity of many dog breeds is predicated on them no longer being functional dogs; rather, they are representations or caricatures of human aesthetic desires. The result is breeds in which the teleonome of individual dogs, i.e., the integrated biological system by which they maintain viability has been structurally dismantled in the pursuit of appearance. This article applies teleonomic and allostatic load theory to a multi-domain analysis of extreme brachycephaly as a primary case study, drawing on representative examples from other extreme phenotypes to examine their welfare costs across conformational disorder, innate health, allostatic load, and veterinary costs. A detailed Five Domains analysis of extreme brachycephalic morphotypes, where the epidemiological evidence is most complete, reveals that every major welfare domain is simultaneously compromised from birth because artificial selection has structurally compromised teleonomic function across morphological, physiological, physical, and behavioural traits. Allostatic load theory then provides the mechanistic explanation for the resulting shorter lifespans. The article concludes by addressing the veterinarian’s emerging role as arbiter of animals’ fitness for purpose under consumer and welfare laws, making the case for an Innate Health Assessment to become a standardised functional assessment framework grounded in teleonomic principles.

## Simple Summary

This article applies the teleonome to explain the compounding welfare costs incurred when artificial selective breeding for ‘extreme phenotypes’ prioritises aesthetically driven form over function. The Five Domains Model and allostatic load theory are drawn on to examine the consequences. The term ‘extreme phenotypes’ encompasses what is also described as extreme morphotype, extreme conformation, hypertype, and extreme body shape in regulatory and breed-standard literature, while additionally capturing behavioural phenotypic variants. Together, the three frameworks reveal that the welfare harms of extreme phenotypes in companion animals are not simply clusters of breed-associated disorders; rather, they are manifestations of systemic disruption of evolved biological functions that precipitate suffering and limit opportunities and capacities for positive affect and longevity. For each extreme phenotype popularised by humans, anthropocentric selection prioritised animal traits valued by people while systematically compromising critical biological functions that matter most to the animals themselves. Artificial selection for extreme phenotypes is one of several compounding harms generated by the purebred dog breeding system; others include progressive narrowing of genetic diversity and the entrenching of institutional forces that have discouraged corrective breeding to restore critical function effectively and efficiently. This article focuses on the welfare consequences of brachycephaly in dogs, while acknowledging that a full account of the legacy of selective breeding overall will need to address other consequences.

## 1. Introduction

The teleonome is a biological construct that captures the organisational logic and adaptive capabilities of living beings [1,2]. In sentient animals, it reveals the deep and evolutionarily driven integration of their body systems and related affective experiences seen as a named functional whole, such that any disruption of survival and reproductive capabilities reliably produces suffering. Fundamentally, the teleonome is a product of evolution by natural selection, which refined the perceptual, neurophysiological, morphological and social demands of animals to match the demands and opportunities of ancestral environments. An individual animal’s teleonome does not inhabit that ancestral world, but it still belongs to it in a constitutive sense. Its teleonomic traits encode the potential to navigate the challenges and exploit the opportunities that the animals’ world used to offer, alongside some degree of flexibility to respond to novel demands [3]. The genetic passage of teleonomic traits from one generation to the next is an ecological gamble that assumes the ecological conditions the offspring will encounter will resemble those that shaped their ancestors. When that gamble fails—because the environment shifts too radically, or when ill-advised human intervention corrupts teleonomic integrity, the teleonome can no longer support viability. In extreme brachycephaly in dogs, the gamble was decisively lost at the point when these dogs’ morphology rendered them unable to breathe, blink, sleep, exercise, or reproduce to the level of a typical dog [4,5,6,7,8,9,10,11,12]. Selective breeding that prioritises form over function has produced animals whose teleonomic architecture has been hugely distorted; thus, the ancestral configuration of physical and behavioural capabilities is unsuited to the modern animals’ circumstances, due explicitly to human demands for extreme morphologies.

Such dismantling of the canine teleonome has broad welfare consequences because to maintain viability and pursue biological goals, the teleonome operates continuously through the physiological, behavioural, and adaptive bio-regulatory processes that generate affective experiences fundamental to welfare states.

### 1.1. The Teleonome, the DEFR+U Cycle, Affordances, and Allostatic Load

These teleonomic processes have been conceptualised as cycles of *detection*, *evaluation*, *forecasting,* and *response*, followed by *updating* priors (DEFR+U) [3]. Affective states arise within this cycle as integral and dynamic signals that motivate and govern behaviour [13]. Negative affect signals a deviation that requires resolution, and positive affect signals that regulation is proceeding toward resolution. The Five Domains Model provides the welfare framework within which these cycles operate, while the teleonome explains why particular circumstances generate specific motivations and affective states, and why particular responses matter to the animal. When regulatory challenges can be successfully resolved, the cycle updates future expectations in ways that support the animal’s adaptive functioning, thereby building competence and resilience and supporting welfare. When resolution is prevented or protracted, either because appropriate affordances are absent or inaccessible, or because the animal’s regulatory capacity is depleted, negative experience persists, allostatic load (a more detailed discussion is provided in Section 5) accumulates, and welfare is compromised.

The ability of animals to regulate internal conditions depends both on regulatory capacity and on the opportunities for action that their environment affords. These opportunities, termed affordances, arise from the relationship between environmental features and the animal’s physical and behavioural capabilities [14,15]. For example, in warm-to-hot conditions, a functional canine airway affords effective panting and thermoregulation, whereas a structurally compromised airway (e.g., due to extreme muzzle foreshortening) does not. Affordances are therefore properties both of environments and of the interaction between environments and the animal’s capacity to use them to achieve biological goals (telos) [3].

Allostasis describes the physiological and behavioural adjustments through which animals respond to predictable and unpredictable stressors, while allostatic load refers to the cumulative biological cost that is incurred [16]. Under normal conditions, allostatic load is manageable; the system is activated, meets the demand, and returns to the homeostatic baseline. Allostatic overload occurs when that recovery is impaired, either because resolution is protracted (Type 1 overload) or because the challenge is structurally unresolvable. When effector systems are structurally impaired, regulatory demand persists without resolution, generating chronic negative affect that animal welfare science has struggled to describe without a theoretical foundation adequate to the task [3]. In this way, allostatic load and its pathological extreme, allostatic overload, provide a mechanistic explanation for how persistent teleonomic disruption translates into cumulative welfare compromise.

### 1.2. The Affectome and Biologically Relevant Welfare Assessments

The teleonome also encompasses the affectome: the subset of the animal’s teleonomic traits responsible for generating, qualifying, and prioritising affective responses [3,17]. The affectome reflects each animal’s specific evolutionary history, ecological niche, and the particular affordances that their perceptual and motivational systems evolved to exploit. In doing so, this explains why animals are motivated to do what they do, and why thwarting the behaviours they generate produces suffering.

Meaningful welfare assessment therefore requires understanding what a species is biologically equipped, motivated, and afforded the opportunity to do and whether they can effectively act on these motivations. Without that foundation, welfare assessments risk measuring what it is convenient for humans to assess rather than what matters most to the animal. This theoretical backdrop is central to understanding the welfare costs of extreme phenotypes. Artificial selection has altered morphology far more rapidly than the underlying motivational architecture has changed [18]. Consequently, animals with extreme phenotypes may retain a motivational architecture that evolved under ancestral conditions while losing the physical capacities required to fulfil them.

### 1.3. Extreme Phenotypes

Extreme phenotypes have been selected among several domesticated species, but domestic dogs provide some of the clearest examples of teleonomic disruption. Dogs retain an original evolutionary inheritance shaped by selection for capacities including detecting prey by scent, regulating body temperature across a range of climates, sustaining locomotion over varied terrain, coordinating with conspecifics, and unassisted reproduction. Together, these and other evolutionarily conserved capacities form an integrated biological system through which dogs maintain viability [3]. Extreme brachycephaly in dogs illustrates how rapidly artificial selection can compromise that system. Affected dogs may be unable to breathe, sleep, exercise, communicate, or thermoregulate even under domestic conditions with abundant environmental affordances, without extreme demands or stressors being placed upon them. Their welfare challenges arise primarily from inherited structural limitations explicitly selected by humans rather than arising from deficient management or unsuitable environments, because their own bodies prevent timely completion of the functional loops that constitute normal canine life. Accordingly, the teleonome is not merely constrained; in the most extreme cases, it is, as our title suggests, in tatters.

### 1.4. Recognising Extreme Phenotypic Problems and Doing Little About Them

The negative consequences of extreme canine phenotypes were both foreseeable and foreseen. In Darwin’s *The Variation of Animals and Plants Under Domestication* (1868), he discussed the proclivity of humans to propagate morphological mutations in domestic dogs: “Some of the peculiarities characteristic of the several breeds of the dog have probably arisen suddenly, and, though strictly inherited, may be called monstrosities; for instance… the shape of the head and the under-hanging jaw in the bull- and pug-dog…A peculiarity suddenly arising, and therefore in one sense deserving to be called a monstrosity, may, however, be increased and fixed by man’s selection.” Concerns regarding the health impacts of the extreme phenotype of the Bulldog followed in the early 1900s from breeding communities, noting that ‘certain typical points’ were ‘intensifying predispositions to disease’ and producing ‘cripples and deformities’ with ‘a sadly shortened duration of life’ [19]. Nearly three decades before the epidemiological evidence reviewed in this article had accumulated to its present scale, McGreevy and Nicholas [20] identified five structural problems in pedigree dog breeding: (1) breed standards running counter to welfare; (2) insufficient selection pressure on health-relevant traits; (3) unacceptably high incidences of inherited defects; (4) inbreeding driven by small breed populations; and, critically, (5) financial disincentives for veterinarians to reduce the prevalence of inherited diseases, because they are paid to diagnose and treat them. McGreevy’s [21] paper called explicitly for the opposite pathway, i.e., advocating for breeding directed at promoting quality of life and functional health rather than conformity to aesthetic standards that had ceased to serve any biological purpose. As a necessary first step, that paper proposed epidemiological surveillance of the prevalence of inherited disorders, outlining what would later develop into the VetCompass approach, a primary care clinical data collection that has provided strong evidence for the negative predispositions of Bulldogs and other dogs with extreme conformations [22,23,24]. In short, at the turn of the twentieth century, health implications of conformational exaggeration had already been articulated, but that evidence alone was insufficient to alter the trajectory of breeding practices. That failure has allowed the gradual intensification of extreme traits that underpin many of the welfare challenges discussed in more detail in Section 4.

### 1.5. Breadth of Extreme Conformational Problems in Domesticated Species

Before narrowing our analytical focus specifically to brachycephalic dog breeds, it is important to establish that the breadth of conformational types in which extreme selective morphotypic breeding has produced heritable, predictable functional deficits extends to numerous other breeds and indeed to other species.

Across domesticated species, artificial selection has repeatedly exploited behavioural, morphological, and physiological traits that are appealing or profitable to humans at the direct cost of teleonomic coherence in the selected animals (see Table 1). The welfare compromises of morphological dysfunction cannot be remedied by improved care or management alone, since the dysfunction is expressed at rest and while performing species-typical behaviours, in ordinary domestic conditions, and across the full lifespan; it is also an inherited feature that is transmitted to every subsequent generation. The natural-mating difficulties seen in broad-breasted commercial turkeys (Table 1) have a direct parallel in extreme brachycephalic dogs, in which whelping is similarly and predictably compromised (see Table 2, Reproductive row, and Section 3.1).

### 1.6. Brachycephaly in Dogs and the Demand for Malformed Animals

For the purposes of this article, brachycephaly is defined as foreshortening of the facial skeleton relative to the mesocephalic (normally proportioned) canine skull, such that the muzzle is markedly reduced relative to cranial length. Extreme brachycephaly denotes the severe end of this continuum, at which the resulting structural compromise is sufficient to impair one or more basic biological functions, such as breathing, thermoregulation, sleep, or parturition, under ordinary domestic conditions rather than only under exertion or environmental extremes. Mild, moderate, and severe brachycephaly (Section 2.1) refer to increasing degrees of this same structural continuum. Section 4 (Table 2) below maps this continuum against each affected functional system and Five Domains location in detail.

Against this broader context, extreme brachycephalic morphotypes in dogs represent the most extensively researched case of teleonomic failure in domestic animals. The quantity and quality of epidemiological evidence now available for these dogs, primarily through the VetCompass Programme of the Royal Veterinary College [22,23] and related studies, provide a robust case for detailed welfare analysis. That analysis here reveals a population in which every major welfare domain is simultaneously and structurally compromised from birth. Allostatic load theory then provides the mechanistic explanation for the approximately four-year deficit in lifespan reported (8.6 versus 12.7 years; [47]).

The conformational welfare crisis in dogs is an artificially selected problem underpinned by commercial incentives to prioritise the aesthetic preferences of purchasers over dog health and longevity [48]. This social license to breed malformed animals has prevailed for over a century and continues to override our human responsibility to select for the functional requirements of the animals themselves [49]. McGreevy [21] proposed a corrective pathway nearly twenty years ago that has been largely ignored by the veterinary and breeding communities. The Innate Health Assessment, which this article showcases, reopens that corrective pathway. It is significant precisely because it is independent of the breed; its functional capacity criteria apply equally to the Dachshund whose vertebral column cannot sustain normal locomotion and the English Bulldog whose airway cannot sustain efficient breathing and thermoregulation. The professional responses that welfare science is now generating are contextualised by the common principle that the teleonome makes explicit, namely that an animal’s welfare cannot be assessed without reference to what that kind of animal evolved to be able to do because “nothing in biology makes sense except in the light of evolution” [50].

### 1.7. Matters Covered in the Following Sections

The article proceeds as follows. Section 2 establishes the VetCompass epidemiological evidence base for extreme brachycephaly. Section 3 applies the Five Domains Model systematically to that evidence. Section 4 maps the structural compromise of evolved bio-regulatory capacity across functional systems. Section 5 develops the allostatic load argument linking the clinical picture to the shortened lifespan. Section 6 makes the case for a standardised functional assessment tool and introduces the Innate Health Assessment as a teleonomic construct. Section 7 (Discussion) addresses the veterinarian’s emerging role as assessor of fitness-for-purpose, and the conflicts of interest that currently suppress the clinical evidence that role requires. Section 8 (Conclusions) synthesises the key findings and opportunities. Finally provided, as an aid memoir is a glossary of terms utilised in teleonomic reasoning.

## 2. The VetCompass Evidence Base: A Population-Level Picture of Structural Harm Due to Brachycephaly

Any analysis of the welfare costs of extreme brachycephaly in dogs must begin with the epidemiological evidence, because that evidence transforms the clinical narrative from a set of anecdotal concerns into a measurable, population-level welfare emergency. This paper presents VetCompass studies on four broad areas: demography, lifespan, overall brachycephalic disorder patterns, and breed-specific disorder patterns, as the quantitative foundation for the teleonomic interpretation of the welfare effects of extreme brachycephaly.

### 2.1. Demographics

Demography describes statistical studies of population structure and is acknowledged to offer key insights into current and projected trends [51]. Demographic analysis of UK general veterinary practice data shows how widespread extreme morphotypes are in the population, reveals how many animals are bearing these welfare costs, and where these trends are heading. A VetCompass demographic study of over 2 million dogs in 2019 identified high UK ownership of extreme brachycephalic breeds, with Shih Tzu (3.0%), French Bulldog (3.0%), Pug (1.8%), Lhasa Apso (1.1%), and English Bulldog (1.0%) among the 21 most common breeds at any age. Among the dogs aged under 1 year in 2019, the French Bulldog was the most common breed (7.0%), likely reflecting its recent and sudden increase in popularity that led to many new puppies with very short life expectancies (Section 2.2) entering the population. More broadly, 17.6% of all dogs were recorded as belonging to a breed with brachycephaly, reflecting its pervasive cultural normalisation. Even more worrying, among these brachycephalic dogs, the degree of brachycephaly was graded as severe in 53.9%, moderate in 34.2%, and mild in 12.0% [52].

### 2.2. Longevity

Reduced lifespan at a population level provides a broad indicator of disproportionate health burden [53]. VetCompass studies consistently demonstrate shorter life expectancy in brachycephalic breeds compared with both crossbred dogs and the wider dog population [29,54]. Among breeds with sufficient data for robust analysis, French Bulldogs (median 4.5 years), English Bulldogs (median 7.4 years), and Pugs (median 7.7 years) rank among breeds with the shortest life expectancy from their first year of life, compared to a median 11.2 years for dogs overall, and 11.8 years for crossbreeds [54]. These data support the conclusion that the allostatic load for breeds with extreme phenotypes imposes a substantial longevity cost.

### 2.3. Overall Disorder Burden

Population-level analyses corroborate this conclusion, showing that brachycephalic dogs experience higher overall disease burdens than non-brachycephalic dogs. After accounting for demographic confounders (bodyweight, sex, neuter status, and insurance status), the median number of disorders diagnosed each year was 24% higher in brachycephalic dogs than in crossbred dogs [8]. Among the thirty most common disorders in this study, brachycephalic dogs had higher odds of eight of the 30 disorders compared with non-brachycephalic dogs, lower odds of two of the 30 disorders, and no significant difference in odds for the remaining 20 disorders. This study grouped 34 breeds as brachycephalic, many of which showed only mild brachycephaly. Despite this, marked disorder predispositions for brachycephaly were still apparent.

### 2.4. Breed-Specific Disorder Profiles

Studies of individual breeds reveal the extent to which extreme conformation shapes disease risk. French Bulldogs, Pugs, and English Bulldogs all show marked predispositions to disorders directly linked to their extreme morphology, including Brachycephalic Obstructive Airway Syndrome (BOAS), stenotic nares, skin-fold dermatitis, corneal ulceration and reproductive dysfunction [9,10,11,55]. Although prevalence estimates vary between studies, these disorders occur at rates substantially exceeding those observed in the wider dog population. These figures almost certainly underestimate the true burden, given that respiratory compromise in affected dogs has become normalised among their owners and the veterinary profession [56].

Taken together, these epidemiological findings demonstrate that extreme brachycephalic dogs are very common, carry more disorders at an overall morphotype level, show concerningly high disorder predispositions at a breed level, and have substantially reduced lifespans. While a heightened disease burden characterises the population, the most prevalent pathophysiological process behind this disease burden, namely inflammation, is examined in Section 5 as a mechanistic driver of the lifespan deficit.

## 3. A Five Domains Analysis of Extreme Brachycephaly

The Five Domains Model [57] provides a structured framework for welfare assessment that moves beyond the static checklist of Brambell’s Five Freedoms [58,59] to model welfare as a dynamic state. The first four physical and functional domains generate inputs that the animal’s perceptual, evaluative, and predictive systems integrate into the final Domain 5 of affective experience. That affective experience matters to animals because it shapes their motivational architecture, orienting them toward opportunities or away from threats, and influences learning. That learning determines whether experience is building or eroding teleonomic competence. A teleonomic framing of the model thus conceptualises mental state not as the mathematical sum of inputs from the other four domains but as an emergent, dynamic construction that is shaped by the animal’s history of bio-regulatory success or failure [3,13].

In the case of extreme morphotypes, the teleonome and the teleonomic welfare assessment process developed by Wilkins et al. [3,13] deepens this picture, because in animals with an extreme morphotype, Domain 5 is the domain in which the animal’s teleonomic cycles of detection, evaluation, forecasting, and response are chronically overloaded. Within this architecture, Nutrition and Health (Domains 1, 3, and 5) generate the interoceptive signals (e.g., pain, illness, and breathlessness) that contribute directly to the affective valence and intensity felt in Domain 5. The Physical Environment (Domain 2) encompasses the ambient conditions animals perceive through evolved sensory systems, which trigger regulatory adjustments (autonomic and behaviourally mediated) in thermoregulation, activation, and other homeostatic adjustments (allostasis). Behavioural Interactions (Domain 4) represents the full breadth of the animal’s behavioural repertoire and motivational architecture. This repertoire includes the animals’ capacity to act on their motivations, avoid threats, and pursue meaningful goals through the affordances the environment provides [3]. Critically, the teleonome clarifies that the Five Domains operate as interactive and relational processes rather than isolated categories. Affective states shape behaviour; behaviour acts on the environment’s affordances to resolve challenges and seize opportunities, and the resulting conditions across the four physical domains feed forward to calibrate future perception, appraisal, forecasting, and action. Mental states are thus not static summaries of the conditions in domains one to four, but dynamic constructions shaped over time by the animal’s ongoing interaction with the world. Extreme brachycephaly provides a particularly informative case study because multiple domains are compromised simultaneously from birth, creating persistent barriers to teleonomically coherent physiological and behavioural regulation.

### 3.1. Domain 1: Nutrition

The nutritional domain in extreme brachycephalic dogs is disrupted through two structurally independent mechanisms, both rooted in the same artificially selected anatomical cause. The elevated negative intraluminal pressures generated during every inspiratory effort in a BOAS-affected dog directly impairs the lower oesophageal sphincter, producing aerophagia, gastro-oesophageal reflux, regurgitation, oesophageal dysmotility, and hiatal hernia as concomitant structural consequences [60,61]. Poncet et al. [62] reported gastrointestinal (GI) tract lesions in 22% of 73 brachycephalic dogs presenting with upper respiratory syndrome at the time of airway surgery. Among dogs presenting with BOAS to a referral teaching hospital, breed-specific prevalences of GI clinical signs were 93% for French Bulldogs, 58% for English Bulldogs, and 16% for Pugs [63].

Obesity is the single most prevalent disorder in Pugs at 13.2%, with Pugs showing 3.9 times the odds of obesity compared to all remaining dogs. This makes the Pug the breed with the highest predisposition to obesity of all dog breeds [11,64,65]. Obesity has been actively selected as a desirable characteristic in Pugs for decades, with the (Royal) Kennel Club (RKC) breed standard requiring Pugs to ‘never to appear low on legs, nor lean and leggy’ until recent revision to remove ‘lean’ from this clause [66]. Obesity is also an inevitable consequence of exercise intolerance: dogs physiologically unable to sustain normal physical activity due to a structurally impaired airway cannot effectively regulate body condition through the energy dissipation mechanisms their species is equipped to deploy. The negative feedback loop compounds because obesity increases the soft tissue mass around the already-compromised airway, worsening BOAS and increasing heat production [5,67]. Teleonomically, the feeding and foraging systems’ motivational architecture remains fully functional; what is compromised is the metabolic consequence of eating in a body that cannot process energy intake through evolved teleonomic channels.

A further nutritional dimension of teleonomic failure in brachycephalic breeds arises from compromised masticatory function. For example, the current RKC breed standard for the English Bulldog requires an extreme morphotype with a ‘lower jaw slightly projecting in front of upper with moderate turn up’ [66]. Hale [68] documents that extreme skull conformation compromises masticatory function, impairing the dog’s ability to chew safely. In an attempt to address these compromises, rather than selecting away from this deleterious conformation, breed-specific diets have been developed with modified kibble shape to make them easier to pick up and chew. When these anatomical constraints restrict chewing capacity, the dog cannot access the putative physiological, behavioural, and digestive benefits of gnawing—among them the reduction in arousal and allostatic load that sustained chewing affords dogs. The inability to fulfil this component of the canine foraging repertoire generates frustration and forecloses one of the few low-cost regulatory channels available to the dog, compounding the Domain 1 deficit produced by exercise intolerance and GI disease. The structural dental consequences (including crowding, rotation, malocclusion, and a loose mandibular symphysis) are addressed as a source of chronic nociceptive load in Domains 3 and 5 below.

### 3.2. Domain 2: Physical Environment

For extreme brachycephalic dogs, ordinary domestic environments constitute genuine physiological hazards because the threshold at which environmental conditions exceed regulatory capacity has been structurally lowered by artificial selection. Davis et al. [69] demonstrated that brachycephalic dogs show significantly greater increases in respiratory rate as ambient temperatures rise compared with those observed in non-brachycephalic controls. Five of 52 brachycephalic dogs and none of 53 controls required withdrawal from a controlled heat tolerance study due to respiratory distress. Supporting these findings, Gallman et al. [70] identified abnormal upper-airway thermal responses in dogs with BOAS during and after exercise, consistent with impaired respiratory heat dissipation and reduced thermoregulatory efficiency. US Department of Transportation data cited by Fawcett et al. [67] show that approximately half of 122 dogs that died in aircraft cargo holds over a five-year period were brachycephalic breeds, a finding sufficient to prompt airline bans. In the UK, the breeds with the highest odds of heat-related illness compared to the Labrador Retriever under primary veterinary care were Chow Chow (×16.6), English Bulldog (×14.0), French Bulldog (×6.5), and Dogue de Bordeaux (×5.3) [71]. These results suggest that heat-related illness should largely be considered a disorder of the extreme morphotype resulting from loss of thermoregulatory capacity to dissipate heat, with 74.2% of all heat-related illness events in such UK dogs reported to be triggered by exertion [72].

The exercise intolerance that prevents normal thermoregulation in extreme brachycephalic dogs is compounded by owner behaviour operating at three levels: dogs physically unable to exercise even when given the opportunity; dogs whose owners restrict exercise out of protective concern for their dog’s airway malformation; and, critically, dogs whose owners chose that breed specifically because they did not want an animal requiring or capable of sustained exercise [48]. The last of these represents an active selection by owners for a welfare-compromised animal type, which is reinforced by marketing and owner-to-owner recommendation of these breeds as suited to ‘apartment living’ [48,73].

The teleonomic implication is that the thermoregulatory component of these dogs’ teleonomes, which evolved to maintain core temperature across a range of environmental conditions, cannot fulfil its function because the effector pathway (panting) requires a patent airway that no longer exists. There is a clear affordance gap between what the motivational architecture is oriented toward and what the animal’s structural features permit. To make this concrete: on a warm day, a dog with a patent airway detects rising core temperature, increases panting rate, dissipates heat effectively, and returns to normothermic body temperatures. Thus, the DEFR+U cycle is completed. A brachycephalic dog in the same conditions attempts the same response, but the structurally narrowed airway limits airflow to below what thermoregulation requires. The effort of attempting to pant generates additional muscular heat. The regulatory demand remains unresolved, and each episode of thermal stress accelerates wear of already-narrowed airway tissues, progressively reducing future thermoregulatory capacity and worsening the structural conditions for the next attempt.

Olfaction is the primary perceptual modality through which dogs detect and evaluate biologically relevant information. Roberts et al. [74] found that an increasing cephalic index (CI) produces a ventral migration of the olfactory bulb independently of brain size, i.e., a positional shift that may alter its connectivity to the limbic and orbitofrontal structures responsible for assigning biological meaning to detected scents. The welfare consequence may therefore not be reduced odour detection as such (the well-documented working-scent capacity of several brachycephalic breeds argues against simple detection failure), but a disruption to the fidelity with which detected information is evaluated for its relevance. Within the DEFR+U cycle, this would implicate the *evaluation* step specifically, i.e., the translation of a detected cue into a response-relevant signal. This remains an anatomical inference rather than a tested functional deficit, but it raises the possibility that selection for extreme skull shape does not compromise what these dogs can smell, rather what they can interpret of what they smell.

### 3.3. Domain 3: Health

Most of the health conditions to which extreme brachycephalic dogs are predisposed are predominantly structural, congenital, hereditary, and inflammatory. The pathophysiological signature identified by O’Neill et al. [64] is particularly revealing. The dominant pathological process in Pugs is inflammation (38.3%), followed by congenital and developmental conditions (15.2%), nutritional disorders (14.7%), and hereditary conditions (10.9%). This is the profile of an animal whose structure continuously generates inflammatory challenge.

Respiratory ill-health is a central concern related to brachycephaly in dogs, but it should be noted that many of the popular extreme brachycephalic breeds carry many other extreme morphotype predispositions, e.g., higher risk of ocular, skin, reproductive, spinal, and other disorders. Fawcett et al. [67] describe BOAS as a progressive disorder in which primary anatomical abnormalities generate progressively increasing resistance to airflow as animals age. Packer et al. [5] demonstrated a strong positive relationship between degree of facial shortening and BOAS risk, but with disease prevalence increasing non-linearly as relative muzzle length decreased. Specifically, BOAS occurred only in dogs whose muzzle length was less than half their cranial length, providing evidence that the severity of respiratory compromise is intrinsically linked to the extremes of brachycephalic conformation. Laryngeal collapse has been documented in dogs as young as 4.5 months [75]. O’Neill et al. [47] demonstrated 3.5 times the odds of at least one upper respiratory tract (URT) disorder in extreme brachycephalic dogs, with Pugs showing the highest individual breed odds ratio at 6.9.

Ophthalmic health is severely compromised in dogs with brachycephaly. The canine cornea is densely innervated by nociceptive afferent axons, and corneal damage causes substantial ongoing pain [6,64]. Packer et al. [6] found that several hallmark features of extreme brachycephaly, including marked muzzle shortening, skin folds, enlarged palpebral fissures, and exposed sclera, were associated with substantially increased odds of corneal ulceration, suggesting that selection for exaggerated facial conformation predisposes affected dogs to a painful and potentially vision-threatening disorder. Compared with crossbred dogs, the breeds in the UK with the highest odds of corneal ulceration were Pug (×19.1), Boxer (×12.1), Shih Tzu (×10.0), English Bulldog (×7.8), French Bulldog (×7.3), and Cavalier King Charles Spaniel (×6.1) [76]. Compared with crossbred dogs, the breeds in the UK with the highest odds of prolapsed nictitating membrane gland were Neapolitan Mastiff (×34.4), English Bulldog (×33.2), Cane Corso (×23.1), Puggle (×14.5), Lhasa Apso (×10.7), American Cocker Spaniel (×10.2), and French Bulldog (×8.2) [77].

Neurological compromise from skull compression produces Chiari-like malformation and syringomyelia [67], with brain disorders recorded in 2.9% and spinal cord disorders in 1.4% of Pugs in a single year. Research into syringomyelia has identified extreme brachycephalic cranial morphology as a major risk factor, with progressive shortening and doming of the skull associated with reduced space for the caudal brain and impaired cerebrospinal fluid flow, supporting the view that the disorder represents a direct consequence of selection for exaggerated head conformation [78,79].

Dental health constitutes a further structural domain of harm. Hale [68] reported that brachycephalic breeds carry elevated risk of periodontal disease precisely because their extreme skull conformation produces dental crowding, malocclusion, and compromised masticatory mechanics. These dental consequences generate chronic nociceptive input from the oral cavity, impair the dog’s ability to chew, and create a self-reinforcing cycle in which reduced masticatory activity accelerates calculus accumulation and the progression of periodontal disease. Periodontal disease, which is itself painful and inflammatory, adds a further independent source of allostatic cost to a Domain 3 profile already dominated by multi-system structural pathology.

### 3.4. Domain 4: Behavioural Interactions

Domain 4 is the domain where the relational organisation of the teleonome is most directly expressed as each animal attempts to enact evolved strategies for engaging with the world [3,57]. Teleonomic agency has been proposed as a function of the animal’s regulatory capacity (physiological and behavioural resources for meeting demand), the match between what they require and what the environment actually affords them, and the structural integrity of the teleonome itself, i.e., whether the body remains capable of enacting what evolution originally shaped it to do [3,13].

Roedler et al. [4] reported that 88% of dogs referred for BOAS surgery had severe exercise intolerance with a mean onset at 1.12 years. Sleep is profoundly disrupted in BOAS-affected dogs: 56% of referred dogs had significant sleep problems, and affected animals adopted compensatory sleeping postures, including sitting upright, elevating the chin, and maintaining objects in the mouth to prop the airway open [4].

The behavioural consequences extend to the motivational architecture itself. By integrating cephalic index data with owner-reported behaviour across 49 breeds, McGreevy et al. [80], found that skull shape covaries with eight distinct behavioural traits. Short-skulled dogs showed significantly higher rates of compulsive staring, allo-grooming, and dog-directed aggression, and significantly lower rates of chasing, food stealing, and stranger-directed fear. Domain 4 is compromised at both the structural and motivational level simultaneously.

McGreevy et al. [81] found that increasing cephalic index replaces the ancestral visual streak, a horizontal band of high-density retinal ganglion cells affording wide peripheral surveillance, with a concentrated central area favouring acuity over breadth, suggesting a structural account for some of the behavioural shifts reported above. A dog with reduced peripheral surveillance capacity is structurally less equipped to complete detection across its full ambient field; the compulsive staring and reduced stranger-directed fear reported by McGreevy et al. [80] may reflect reduced visual acuity in the peripheral visual field, forcing reliance on sustained central fixation rather than an altered temperament. Read alongside Roberts et al.’s [74] report of ventral brain-axis pitching in high-CI (cephalic index) dogs (Section 3.2), this raises the possibility that two independent consequences of selection for skull shape, visual and olfactory reorganisation, compound at the *detection* stage of the cycle. This is an inference from anatomy, not a tested behavioural mechanism.

Domain 4 is not uniformly compromised, however. Some aspects of dog–human communication may be enhanced rather than impaired in brachycephalic dogs. Dogs with a higher cephalic index have been reported to perform better at following human pointing gestures and to show a greater tendency to establish eye contact, and Ujfalussy et al. [82] also found that brachycephalic dogs oriented more towards humans than did mesocephalic dogs. This dog–human interaction component of Domain 4 should therefore be read alongside the deficits documented above, rather than as a uniform picture of behavioural compromise.

The compromised behavioural agency of brachycephalic dogs extends to environmental problem-solving [13]. Ujfalussy et al. [82] found that brachycephalic dogs show longer latency when opening food puzzles, use their paws less during manipulation tasks, and rely more on their human carers than mesocephalic dogs. This pattern of reduced physical and cognitive engagement with the environment is consistent with both the motivational constraints imposed by extreme skull conformation and the documented reduction in exploratory and foraging behaviour in these morphotypes. It suggests that the behavioural deficits are not limited to physically demanding activities such as running; they extend to the subtler manipulative and investigative behaviours through which dogs normally interact with their environment and access enrichment.

A teleonomic reading of Domain 4 reframes it from a repository of behavioural outputs into the domain where the teleonome’s relational organisation is expressed or thwarted. Animals do not execute fixed behavioural routines; they are instead attempting to enact evolved strategies for engaging with the world in adaptive ways. Positive welfare depends on ambient conditions and affordances that support those strategies, i.e., ones that make biological sense to the animal, offering challenges they can resolve, opportunities they can pursue, and outcomes they can learn from to build confidence. This shifts the welfare question from observing what animals do to considering what they are evolutionarily equipped to try to do, and whether affordances enable, constrain, or fail to trigger those attempts [3]. With their stiff vertebral columns, many brachycephalic morphotypes are unable to autogroom, notably the perianal region. Some affected individuals attempt to resolve this disability by rubbing their perineum on household infrastructure [83]. The impact of taillessness on social communication is intuitive but has yet to be reported. However, experimental studies have demonstrated that dogs respond differently to conspecific tail signals depending on tail length, suggesting that taillessness and extreme tail shortening can reduce the visibility and effectiveness of important communicative cues [84]. A parallel constraint operates at the facial level: Schatz et al. [85] report that the musculature used for mimicry of brachycephalic dogs is structurally altered, reducing their capacity to form clear facial expressions, which may help explain why human observers frequently misread the affective signals of extremely brachycephalic dogs [86].

The evidence across all three components of teleonomic agency reinforces a precautionary default to the teleonomic principle: that motivations shaped over evolutionary time are not erased by artificial selection of the generations that represent just 1% of a species’ natural history. Where motivated behaviours appear absent, the parsimonious explanation is that a body that fails repeatedly at resolving a particular bio-regulatory process will cease attempting to do so, or will redirect resources to whichever channels are available, as the compensatory postures, compulsive staring, and carer dependence documented above suggest. The broader picture of disorders and reduced lifespan reinforces this reading. What has come to be accepted as normal behaviour for these morphotypes is more likely to represent teleonomic incoherence rather than successful adaptation to morphological constraints.

### 3.5. Domain 5: Mental State

Domain 5 is important because it is where the cumulative success or failure of biological regulation throughout the lifetime becomes lived experience, contextualising the inputs of the other domains [3]. Layering the regulatory process (DEFR+U) over the Five Domains transforms the model from a static categorisation into a trajectory of adaptive regulation. Domains 1 to 4 represent the inputs and contextual conditions, and the DEFR+U describes how those inputs are transformed into affective experience in Domain 5. Correspondingly, the teleonome dispels a common misconception that Domain 5 contains the other domains as if the mental state were an additive summary of nutritional status, health, environment, and behaviour. Teleonomically, Domain 5 represents the dynamic interaction of those domains, filtered through the animal’s perceptual, cognitive, and motivational systems. Affective experience is not the result of accumulation but of the animal’s own interpretation of the teleonomic trajectory: a constructed and dynamic state shaped by predictive processing [87], perceived behavioural options, and motivational relevance. Welfare, then, represents the affective register of that trajectory and whether it is trending toward regulatory success, competence, and resilience, or toward failure, depletion, and overload. The affectome, as a subset of the teleonome, explains how Domain 5 arises and why it must be the focal point of any welfare evaluation that claims to reflect the animal’s perspective. In extreme brachycephalic dogs, the dominant affective state is one of chronic unresolved demand: the animal detects the problem (e.g., air hunger, pain, and overheating), evaluates it as a viability threat, and attempts to respond, but the response system is structurally blocked.

**Figure 1:**
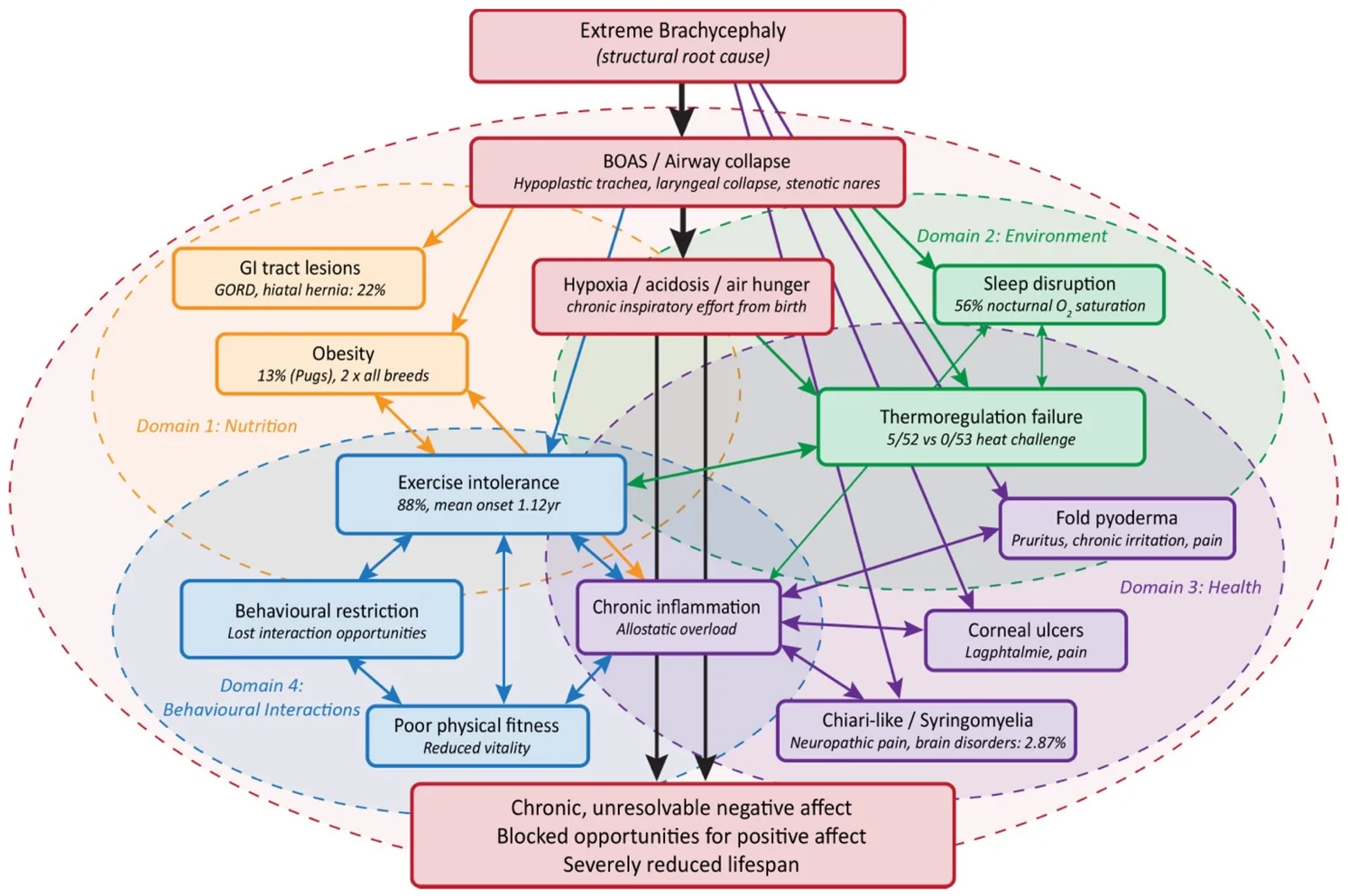
Schematic cross-domain cascade and Five Domains overlap in Pugs. Dashed ellipses indicate the Five Domains of Welfare [57]; nodes positioned within overlapping zones represent conditions that simultaneously compromise more than one domain. Extreme skull foreshortening is the single structural root cause common to all cascades. Illustration by Cristina L. Wilkins.

Arguably, the most important affective state arising from the canine brachycephaly literature is air hunger. Beausoleil and Mellor [88] argue for breathlessness as a distinct and seriously underappreciated animal welfare issue, noting that air hunger is consistently rated in human experience as more aversive than somatic pain of equivalent perceived intensity. The two are not commensurable in kind, since air hunger involves the apprehension of suffocation rather than nociception, but both are aversive, and the available evidence places air hunger as the more distressing of the two experiences. This aligns with the teleonomic logic of affective triage: a threat that is both immediately lethal and potentially resolvable must generate maximal urgency. In BOAS-affected dogs, air hunger is not episodic but constitutes a chronically elevated baseline that reduces the energetic and attentional resources available for other tasks [13]. The extent to which BOAS constrains normal activities is further illustrated by reports that, following surgery to alleviate some aspects of airway obstruction, owners commonly described improvements in exercise tolerance, endurance, sleep quality, and overall quality of life, suggesting that respiratory obstruction directly limits these aspects of daily functioning (Roedler et al. [4]). Studies have demonstrated that brachycephalic dogs experience more disturbed sleep than non-brachycephalic controls and that sleep quality deteriorates with increasing BOAS severity, consistent with the development of sleep-disordered breathing analogous to human obstructive sleep apnoea [12,89]. Sleep deprivation amplifies many other Domain 5 inputs: an animal that cannot maintain normal sleep patterns cannot restore the regulatory systems that process the aversive inputs from Domains 1 through 4 [90]. Using a schematic approach with a focus on Pugs, Figure 1 shows how disorders affect the function of various systems in a cascade.

## 4. Structural Compromise of Evolved Bio-Regulatory Capacity: A Systematic Mapping

The Five Domains analysis that follows uses extreme brachycephaly in dogs as its detailed case study. The analytical approach applies across all extreme phenotypes surveyed in Section 1, but the epidemiological evidence is most complete for brachycephalic dogs, making them the most appropriate focus for this level of domain-by-domain detail. A comparable analysis for other extreme phenotypes awaits equivalent epidemiological depth and represents a priority for future work.

Table 2 synthesises a Five Domains analysis for extreme brachycephaly in dogs for the current paper, mapping each functional system against its evolved regulatory capacity, the specific structural compromise imposed by extreme brachycephalic morphology, the key evidence, its Five Domains location, and its allostatic consequence.

**Table 2:** Structural compromise of evolved regulatory capacity in extreme brachycephalic dogs: a systematic mapping across the Five Domains of Welfare, with key evidence and allostatic consequences.

Functional System	Evolved Regulatory Capacity	Structural Compromise from Selection	Key Evidence	Five Domains Location	Allostatic Consequence
Respiratory/Thermoregulatory	Panting as primary heat dissipation; airway as the effector of the thermoregulatory response	Stenotic nares, elongated soft palate, hypoplastic trachea, aberrant turbinates block airflow	3.5× odds of URT disorder [47,71,72,91,92,93,94,95]; 8.6 vs. 12.7 yr lifespan gap; 5/52 brachycephalic dogs withdrew from heat challenge vs. 0/53 controls [67]; stenotic nares and BOAS are highly inherited traits in these breeds [96]	Domains 2, 3, and 5 (environmental thresholds for thermoregulation exceeded; ordinary ambient conditions constitute physiological hazard; air hunger as chronic negative affect)	Continuous HPA and sympathetic activation at rest; ordinary ambient conditions constitute physiological hazard
Respiratory/Gas Exchange	Efficient airway mechanics; CO_2_ clearance; acid-base regulation	Chronic inspiratory resistance produces respiratory acidosis, hypoxia, and air hunger as resting baseline	88% exercise intolerance, mean onset 1.12 yrs [4]; 56% sleep problems; 10.4% URT disorder in Pugs in one year [64]; increasing brachycephaly (facial flattening) elevates BOAS risk [6]	Domains 3 and 5 (air hunger as chronic negative affect)	Acidosis; cellular hypoxia impairs repair; metabolic cost of breathing elevated continuously
Gastrointestinal/Nutritional	Oesophageal sphincter integrity; peristaltic regulation; gastric acid management	Chronic inspiratory effort generates negative intraluminal pressure displacing the gastro-oesophageal junction	22% GI signs in BOAS surgical patients [62]; enteropathy 11.2% in Pugs [97,98]; digestive system affected 25.5% [64]	Domains 1, 3, and 5	Aerophagia; chronic nausea and reflux as persistent aversive interoceptive signals; impaired nutrient absorption under chronic inflammation
Dental/Masticatory	Mastication as the mechanism for tooth wear, gingival stimulation, and periodontal health; chewing as a restorative, calming behaviour mediated by dopamine and serotonin pathways [99]; jaw muscle function and mandibular alignment	Extreme skull foreshortening crowds 42 teeth into a reduced jaw space, producing dental crowding, tooth rotation, malocclusion, traumatic buccal granulomas, and under-erupted teeth [68]; shortened muzzle reduces jaw muscle engagement, impairing chewing mechanics and the restorative function of mastication	Hale [68]: brachycephalic breeds face elevated periodontal disease risk; Ujfalussy et al. [82]: brachycephalic dogs use altered manipulative behaviours; Quinn et al. [99]: chewing provides a calming, restorative welfare function	Domains 1, 4, and 5	Chronic periodontal pain adds nociceptive allostatic load; loss of chewing as a restorative, calming behavioural channel removes an important regulatory mechanism; impaired food processing reduces nutritional efficiency under chronic inflammation
Metabolic/Body Condition	Exercise as the primary mechanism for energy balance regulation	Exercise intolerance prevents normal energy expenditure; obesity worsens BOAS in a compounding cycle	Obesity most prevalent single disorder in Pugs at 13.2% vs. approximately 6% across all breeds [11,64,65]; body condition score (BCS) positively associated with body temperature [67]; increased BCS risk factor for BOAS [6]	Domains 1 and 3	Obesity-worsened respiratory compromise compounds allostatic load; metabolic syndrome risk elevated
Ophthalmic/Sensory	Corneal integrity; blink reflex; olfaction as primary perceptual modality	Lagophthalmos from exophthalmic morphology; incomplete blink exposes cornea to chronic desiccation and trauma; nasal passage compression impairs olfaction	Ophthalmological disorders 16.3% in Pugs; corneal disorder 8.72% [38,77,100] absent from top 20 across all breeds [64]; brachycephaly and associated anatomical features (increased palpebral fissures, nasal folds) increased risk of ulceration [5]	Domains 3 and 5 (corneal nociceptive input; olfactory deprivation)	Continuous pain signal from densely innervated corneal tissue; immune-mediated secondary disease from chronic inflammation
Integumentary/Dermatological	Skin barrier function; absence of deep fold anatomy in ancestral form	Facial and body skin folds create chronic warm, moist environments for bacterial and fungal proliferation	Dermatological disorders 15.6%; integument most common organ system 31.8% [40]; inflammation dominant pathophysiological process 38.3% [64]	Domains 3 and 5 (pruritus, pain)	Inflammatory cytokine load from chronic skin infection adds to systemic allostatic burden
Neurological/Musculoskeletal	Normal skull-to-brain volume ratio; patent CSF flow; vertebral column integrity	Skull compression forces brain deformation (Chiari-like malformation, syringomyelia); hemivertebrae cause spinal instability and neuropathic pain	Brain disorders 2.9%; spinal cord disorders 1.4% in one year in Pugs [33,64]; Chiari-like malformation documented [67]	Domains 3 and 5 (neuropathic pain)	Neuropathic pain is a high-magnitude, persistent allostatic stressor with poor response to normal canine coping behaviours
Behavioural/Agency	Exercise, exploration, olfaction, and social behaviour as primary modes of affective regulation	Exercise intolerance, sleep disruption, olfactory impairment, and altered motivational architecture constrain the full behavioural repertoire	88% exercise intolerance; 50% heat sensitivity; sleep compensations; skull shape covaries with behaviour [4,80]; heat-related illness risk elevated [71,72]. Exercise intolerance operates at three levels: dogs physically unable to exercise; owners restricting exercise protectively; and owners who selected these breeds specifically because they did not want a dog capable of sustained activity.	Domains 4 and 5	Loss of exercise as an allostatic regulatory mechanism; arousal cannot be resolved through normal canine behavioural channels
Sleep/Restorative	Sleep as the primary window for HPA restoration, immune function, and cellular repair	Upper airway obstruction prevents normal sleep posture; nocturnal desaturation likely; compensatory postures required	56% sleep problems in BOAS referral population [89,101]; dogs sleep sitting up, with head elevated, frequent position changes or sleeping with objects in mouths [4,7]	Domains 4 and 5	Sleep deprivation is the most powerful amplifier of allostatic load; prevents restoration of all other stressed regulatory systems
Reproductive	Independent parturition; normal neonatal survival	Extreme brachycephaly renders natural whelping impossible; elective caesarean section required as a routine necessity	Documented as routine in Bulldogs and French Bulldogs [102,103]; caesarean sections excluded from pet insurance as expected elective procedure [67]	Domain 3	Reproductive dependency on human surgical intervention represents collapse of a viability-critical capability; heritable transmission of structural compromise to offspring

BOAS (brachycephalic obstructive airway syndrome), HPA (hypothalamic–pituitary–adrenal), URT (upper respiratory tract), GI (gastrointestinal), CSF (cerebrospinal fluid).

Every system compromise generates what is anecdotally labelled wear-and-tear (see allostatic cost, Section 5), and none of the challenges listed above is episodic or even partially resolvable without surgical or environmental intervention. The compromises are structural and permanent, are present from birth, have an impact even when dogs are at rest, and generally compound across the dog’s lifespan. In light of this, some of the accumulated consequences of compromised welfare have been indicated in conceptual frameworks, notably the Welfare-Adjusted Life Years model [104], which includes the calculated impacts of impaired welfare and abbreviated lifespan.

## 5. Allostatic Load as the Mechanism for Shortened Life

Allostasis is the process by which organisms achieve stability through change: the anticipatory and reactive adjustment of biological systems to predicted and unpredictable demand, both environmental and internal [105,106]. Under typical resolvable biological conditions, allostasis is adaptive; it shifts the homeostatic operational range to fulfil increased demand and restores the homeostatic baseline as conditions ease. However, allostasis is costly and, when challenges are chronic, multiple, or unresolvable, allostatic load accumulates. McEwen and Stellar [106] established allostatic load as the mechanism by which chronic stress translates into pathological change (allostatic overload) across multiple biological systems.

### 5.1. Allostasis Within the Five Domains Model

Situated within the Five Domains framework and from the teleonomic perspective, allostasis links physiological and behavioural adaptation across all four input domains. Biomarkers of Nutrition and Health (Domains 1 and 3) reflect the internal allostatic state most directly, expressed in circulating concentrations of hormones, blood glucose, metabolites, and inflammatory markers. The Physical Environment (Domain 2) represents the external sources of allostatic demand, i.e., the external physical and changing conditions against which the animal’s regulatory systems must work. For animals with sufficient thermoregulatory capacity, ordinary domestic conditions impose minimal load. In contrast, for extreme brachycephalic dogs, the same conditions comprise a physiological hazard: the structural compromise of the panting pathway means that the environmental demand chronically exceeds the regulatory capacity available to meet it. Behavioural expression (Domain 4) reflects motivational trade-offs and energy allocation shaped by those internal states. Mental State (Domain 5) represents the animal’s lived experience in terms of how well the teleonome is managing simultaneous and competing regulatory demands. Within this framing, allostatic load is the cumulative cost of repeated attempts at adaptation across all five domains simultaneously. Its accumulation can be estimated and graded through a wide range of biomarkers spanning endocrine, immune, metabolic, and neurophysiological measures, offering a biologically grounded basis for monitoring cumulative welfare compromise over the animal’s lifespan [107,108].

While allostatic load has become a cornerstone of human stress medicine, its translation to veterinary clinical practice has regrettably been very slow. Edes et al. [107] demonstrated that allostatic load is as applicable to non-human animals as to humans, and that composite biomarker indices can predict morbidity and mortality in captive populations. However, a scoping review by Seeley et al. [16] found a marked lack of uniformity in how allostasis is conceptualised and measured across animal species, and noted that validated, species-specific allostatic load indices remain largely absent. Structural barriers compound this. For example, veterinary training has tended to be organised around acute pathophysiology rather than cumulative burden, veterinary consultations are episodic and complaint-driven, and even the stress of venepuncture itself may confound neuroendocrine monitoring [109]. Behavioural indicators of chronic stress are frequently normalised by owners or attributed to temperament, particularly when behaviour-specialist referral pathways are difficult to access [110]. Frameworks such as the Five Domains Model [57] and the teleonome [3] offer conceptual scaffolding for longitudinal, affect-centred clinical reasoning.

In extreme brachycephaly, the allostatic challenge is not imposed by the environment but by the body itself. From birth, every major physiological system carries structural load simultaneously:

- Respiratory: increased work of breathing at rest, progressive airway occlusion [111];
- Thermoregulatory: operating at capacity under ordinary ambient conditions;
- Gastrointestinal: chronic negative intraluminal pressure from inspiratory effort;
- Neurobiological: concurrent pain from dental, ophthalmic, dermatological, and spinal sources;
- Sleep: physiologically non-restorative, for which compensatory postures are required;
- Reproductive: natural whelping rarely possible; caesarean section a routine necessity.

Each of these is a distinct and cumulative source of allostatic load, and it extends the welfare effects beyond physiology to include the behavioural, cognitive, and affective sequelae of chronic regulatory challenge. The contemporaneous presence in the same animal means their welfare effects are not simply additive. Chronic hypoxia impairs cellular repair [112]. Chronic sleep disruption elevates pro-inflammatory cytokines and impairs immune surveillance [113]. Chronic HPA activation accelerates cellular senescence [114]. Chronic systemic inflammation, present in 38.3% of Pugs in any given year [64], maintains the microenvironment in a state that accelerates atherosclerotic change, promotes tumour development, and impairs tissue damage resolution [115].

**Figure 2:**
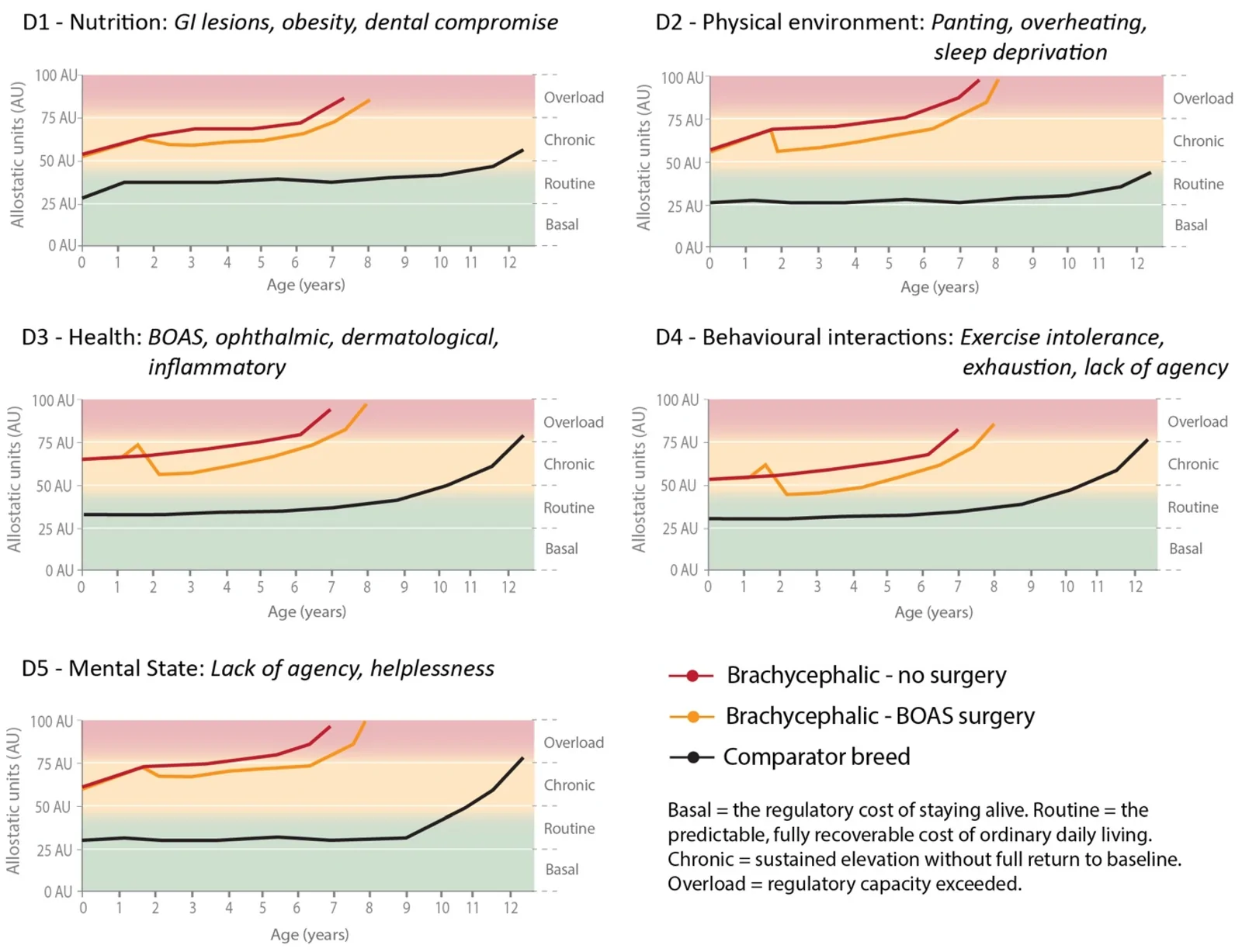
Schematic illustration of cumulative allostatic load in extreme brachycephalic and comparator dogs across the lifespan and across five welfare domains (Five Domains Model: [57]). Cumulative allostatic load across the lifespan in comparator and extreme brachycephalic dogs, shown separately for each of the Five Domains of Welfare [57]: Nutrition (D1), Physical Environment (D2), Health (D3), Behavioural Interactions (D4), and Mental State (D5). D5 is the integrative zone that represents the dog’s agency and teleonomic competence (comparator) or lack of agency and helplessness due to chronic regulatory challenge across the lifetime (brachycephalic). Allostatic units are schematic and proportional, calibrated to be consistent with VetCompass disorder prevalence data [64] and the clinical profile reported by Fawcett et al. [67]; they do not represent a validated composite instrument. Illustration by Cristina L. Wilkins.

The longevity gap documented in O’Neill et al. [47] is the predicted biological consequence of accumulating disproportional amounts of allostatic load from birth [54]. O’Neill et al. [29] note that the Bulldog median longevity of 8.4 years against the crossbred median of 13.1 years represents a gap of approximately one-third of the comparator group’s lifespan. Teleonomically, this gap is the measurable record of what it costs an animal to attempt to maintain viability against morphological constraints it cannot resolve across the Five Domains (Figure 2).

Figure 2 makes the epidemiological data visible: extreme brachycephaly is not a respiratory disease with some complications but a whole-body trajectory consistent with Type II (chronic, unresolvable) allostatic overload from birth. Chronic overload of this kind causes wear and tear and progressively depletes the bio-regulatory capabilities that would otherwise buffer against periods of increased demand and unpredictable challenges [116,117]. This makes Type I overload (the emergency life-history event that is triggered by a precipitating demand the system can no longer meet) both increasingly likely and increasingly lethal as the animal ages. The longevity gap is the population-level signature of the extreme brachycephalic welfare trajectory, indicating that dogs exhausted by chronic overload are not able to resist an unpredictable or acute perturbation that the comparator normocephalic dog would resist and recover from. The post-BOAS surgery trajectory reduces the severity of Type II overload in the respiratory and behavioural domains but does not restore the regulatory capacity to the level of the normocephalic dog, and welfare compromises remain in all the domains.

### 5.2. The Costs of Managing Allostatic Load

**Figure 3:**
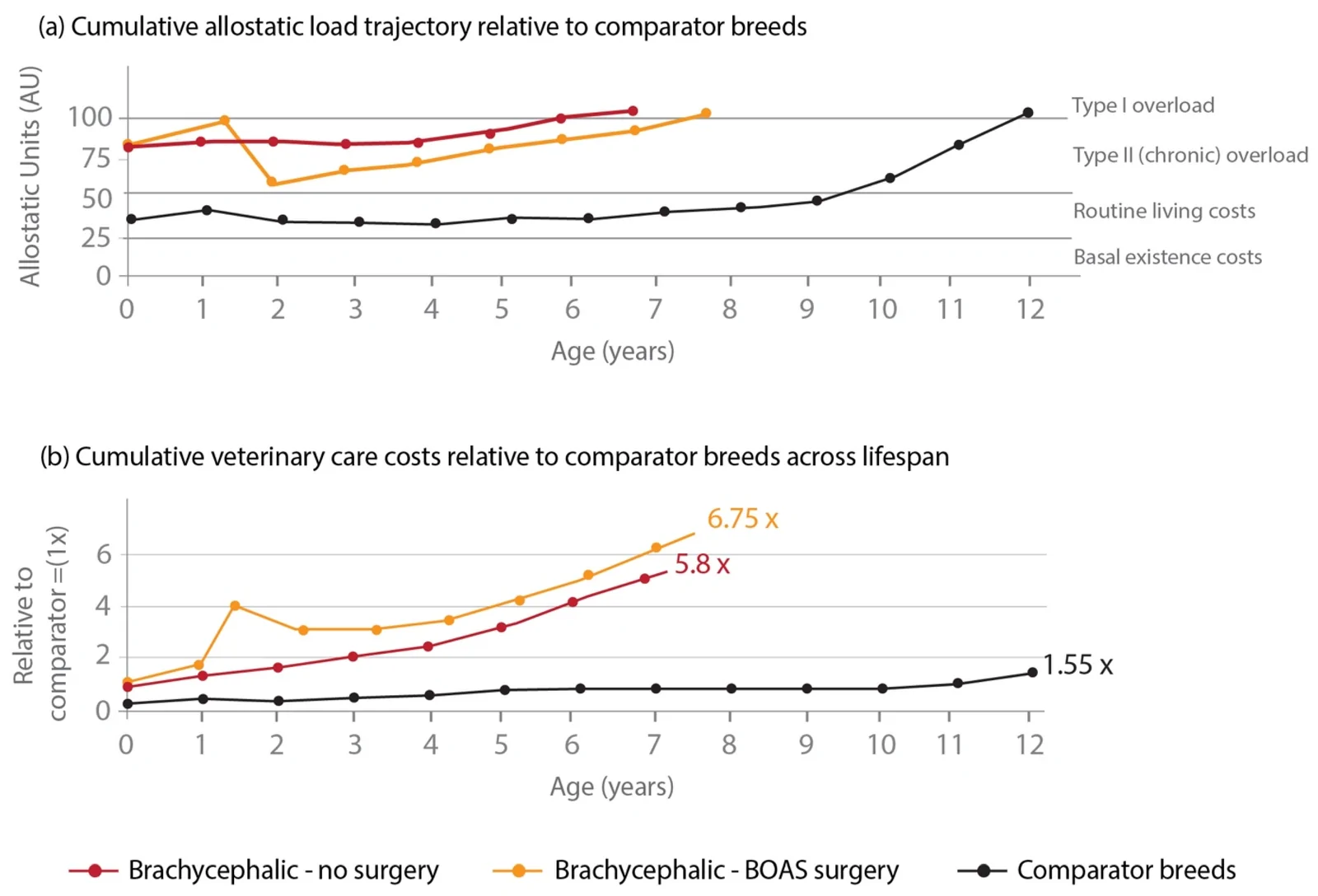
Schematic lifespan comparison of dogs with severe brachycephaly without BOAS surgery (orange), those with severe brachycephaly with BOAS surgery (red), and of comparator dogs free of BOAS (black): Panel (a) the cumulative allostatic load, and Penel (b) the relative cumulative veterinary care costs. The lifetime trajectories are proportional and illustrative, calibrated to be consistent with published VetCompass disorder prevalence and longevity data [47,64,67]. Panel (**a**) shows brachycephalic dogs that begin life with allostatic load already in the Type 2 (chronic, irresolvable) in the overload zone, showing that BOAS surgery reduces the load transiently but does not restore regulatory capacity to comparator dog levels. The trajectories of the two BOAS groups terminate well before the lifespan of the comparator group. Panel (**b**) shows the cumulative economic cost of these allostatic burdens relative to the comparator breeds. Population-level longevity data for surgically managed brachycephalic dogs are not yet available from primary care studies; the post-surgery trajectory is therefore illustrative. Allostatic units (AU) are proportional and schematic, not a validated composite measure. Teleonomic framework: Wilkins et al. [3]. Illustration by Cristina L. Wilkins.

Figure 3 provides schematic allostatic load trajectories of comparator breeds, extreme brachycephalic dogs without surgical intervention, and extreme brachycephalic dogs following BOAS surgery. The trajectories are calibrated to be consistent with published VetCompass disorder prevalence and longevity data [47,64,67]. The brachycephalic dog begins life with all Five Domains simultaneously loaded [approximately 50 Allostatic Units (AU) at birth]. The comparator dog carries a low, slowly rising burden throughout its lifespan. The post-BOAS surgery trajectory reflects the partial relief conferred by surgical correction of upper airway obstruction: respiratory allostatic cost is reduced, but ophthalmic, neurological, dermatological, dental, and reproductive conformational burdens persist, and the underlying morphological cause is not resolved.

The cumulative veterinary cost trajectories are proportional and illustrative; absolute values will vary by practice and jurisdiction. Relative burden is consistent with VetCompass disorder prevalence data showing that extreme brachycephalic dogs carry more simultaneous disorders, present more frequently, and require more surgical interventions than comparator breeds [64,67]. The post-BOAS surgery trajectory includes the surgical episode and subsequent management of conformational domains unaddressed by airway surgery. Costs are normalised to the comparator lifetime total (1×).

## 6. Towards a Standardised Functional Assessment: The Innate Health Assessment

The need, identified in Section 7.3, for a framework to assess whether an animal’s morphotype allows them the capacity to perform their basic biological functions has recently been addressed in the UK through the development of the Innate Health Assessment (IHA) [118]. Launched by the All-Party Parliamentary Group for Animal Welfare in November 2025, the IHA is a publicly available functional assessment framework that evaluates whether an individual dog possesses the capacity to perform ten observable functions considered essential to companion animal welfare.

The concept of innate health holds that all animals, regardless of type or breed, should be capable of performing all the typical activities necessary for their wellbeing within the anatomical, physiological, and behavioural evolutionary norms for the species and the animal’s stage of life. The IHA operationalises this concept through ten binary criteria spanning respiratory, thermoregulatory, locomotory, ocular, reproductive, integumentary and sleep-related domains (e.g., Can *the animal breathe freely without audible effort at rest? Can the animal thermoregulate without distress under ordinary conditions*?). The assessments are observational rather than diagnostic, requiring no specialist equipment and permitting application by owners, breeders, general practitioners, inspectors, or appropriately trained assessors.

A key strength of the framework is its focus on functional capacity rather than breed designation or morphometrics. The IHA is breed-independent, assessing inherited welfare-relevant deficits associated with morphotype irrespective of whether they occur in brachycephalic, chondrodystrophic, or any other conformational type. This approach avoids the limitations of breed-specific regulation, which remains vulnerable to the emergence of new conformations or the reclassification of existing breeds. The relevant welfare question is not whether an animal belongs to a particular breed, but whether its inherited conformation compromises its ability to perform biologically normal functions.

Conceptually, the IHA represents an operationalisation of the teleonome. Each criterion evaluates whether a welfare-relevant component of the animal’s evolved functional repertoire remains achievable. Failure of a criterion indicates disruption of a biological function that would ordinarily be expected in a healthy member of the species. The framework therefore translates the abstract concept of teleonomic integrity into observable and assessable outcomes.

Its emphasis on observable functional outcomes directly addresses a longstanding challenge in companion animal welfare regulation: the absence of an operational definition of what it means for an animal to be physically fit. By defining fitness in terms of demonstrable functional capacity, the framework provides a practical mechanism for assessing whether selective breeding has compromised welfare and, in doing so, offers an implementable expression of the quality-of-life-oriented breeding principles advocated by McGreevy [21].

## 7. Discussion

### 7.1. The Emerging Legal and Regulatory Context

Until recently, the welfare costs of extreme brachycephaly were primarily a matter of professional and ethical concern within veterinary medicine [119]. That landscape is changing. Consumer protection legislation in multiple jurisdictions, including the Australian Consumer Law [120] guarantee of acceptable quality, creates enforceable entitlements for purchasers of companion animals that are defective at the point of sale. Animal welfare legislation in the United Kingdom [121] and NSW prescribes that a dog or cat used for breeding must be physically and mentally fit, healthy, and free of disease at the time of being mated [122] (clause 10.1.1.3). Case law is beginning to operationalise these standards: in Taylor v Lay [2023] [123], the NSW Civil and Administrative Tribunal found that an English Bulldog puppy diagnosed with severe brachycephalic obstructive airway syndrome and a severely hypoplastic trachea failed the Australian Consumer Law acceptable quality guarantee.

The convergence of consumer law, welfare legislation, and professional conduct obligations is producing a new functional demand on veterinarians: an increasing need to document, record, and disclose conformational disease findings in a manner adequate to support enforcement, regulatory, and judicial proceedings. This is a qualitatively different role, requiring that clinical findings be generated and communicated in a form that is reproducible, legally defensible, and capable of answering the specific question regulators and courts need answered.

### 7.2. The Conflicts of Interest That Suppress the Evidence

The veterinary profession’s capacity to fulfil this role is currently undermined by *structural conflicts of interest* that operate at several levels: financial dependence on the income generated by treating conformational disease; the professional and social pressure to accommodate clients who are attached to their animals and to the breeds they have chosen; the direct personal conflicts of interest of veterinary professionals who themselves own, breed, or show animals with extreme phenotypes; and the embeddedness of parts of the profession within the kennel club institutional ecosystem whose standards and practices are the direct source of the problem. Each of these conflicts operates independently; collectively, they create the institutional inertia that McGreevy [21] identified nearly two decades ago and that has yet to be structurally addressed. Quain et al. (2021) [119] articulated the same mechanism with renewed specificity:

“Veterinary professionals who draw an income from involvement in breeding or treating dogs with extreme brachycephalic conformation have a potential conflict of interest between the interests of the animal and their own interests in drawing an income. This concern underscores the need for veterinary professional associations to proactively educate members of the public about the health and welfare costs of extreme phenotypes, rather than leaving it to the individual veterinarian to act as the moral hero.”

BOAS surgery, ocular procedures, dermatological management, and the ongoing treatment of conformational disease represent significant practice income (see Figure 3). The acceptance of respiratory compromise as ‘normal for the breed’, documented by Fawcett et al. [67] and Packer et al. [7,56] as a pervasive phenomenon among owners and clinicians alike, is reinforced when the profession accepts and perpetuates the same normalisation, such as by offering routine BOAS surgery or elective caesarean sections for extreme brachycephalic breeds. That this conflict of interest was identified in the literature in 2007 and persists in 2026 is a measure of how little structural pressure has been applied to it in the intervening period. There is also a risk of moral injury to the veterinary professionals involved in such remedial and elective procedures and to the dogs’ owners. Pound et al. [124] document this directly, describing UK veterinarians’ pre-purchase consultations for brachycephalic breeds as a recurring source of moral distress, in which clinicians repeatedly find themselves “fighting fires” rather than being able to prevent the underlying conformational harm. While it behoves the veterinary profession as an overall body to lead change, currently there is little evidence of organisation-level leadership even though many individual veterinary surgeons are among the most hard-working and effective advocates for welfare change in relation to extreme conformation in dogs.

### 7.3. Veterinarians as Arbiters of Welfare

If veterinarians are to function effectively as arbiters of fitness for purpose, whether in the context of pre-purchase advice to prospective owners, post-purchase consultation, breeding stock assessment, welfare enforcement support, or consumer law proceedings, a standardised, functional framework for assessing conformational fitness is needed. Such a framework must be sufficiently precise to generate reproducible findings, sufficiently accessible to be applied without specialist equipment, and sufficiently breed-agnostic to span the full range of breeds in which conformational disease is a documented concern. As briefly introduced in Section 7.1, what is needed is a framework that answers the question an owner, a breeder, a regulator, or a court needs to ne answered: ‘Can this animal perform the basic biological functions that are prerequisites for an acceptable quality of life as a companion animal?’ Teleonomically, this is asking whether the animal’s system can, during the lifespan, build teleonomic competence, i.e., effectively resolve or avoid the bio-regulatory challenges that give rise to negative affective states, and maintain sufficient capacity and agency to pursue the goals that give rise to positive affective states.

In its June 2026 *BoardTalk* newsletter, the New South Wales Veterinary Practitioners Board (Australia) [125] set out new professional obligations following Taylor v Lay [2023] [123] NSWCATAP 328, where a bulldog’s BOAS amounted to a “major failure” of acceptable quality under Australian Consumer Law (ACL). Under this NSW guidance, veterinarians must record conformational disease findings with clinical specificity, disclose them to clients without minimising by reference to breed norms, and signpost owners to NSW Fair Trading where disease is likely to have been present at purchase. The Board also endorses the IHA (ten binary criteria spanning five functional categories—respiratory, thermoregulatory, locomotory/ocular, reproductive, and integumentary/sleep-related function; distinct from the Five Domains Model of welfare) as a reference for assessing fitness for breeding under the NSW Prevention of Cruelty to Animals Act (POCTAA) [126] Breeding Code. Financial conflicts of interest do not modify these recording obligations.

The *BoardTalk* approach argues that fitness for purpose is the missing operational concept linking two otherwise undefined legal standards. The POCTAA Breeding Code requires breeding animals to be “physically and mentally fit,” but supplies no functional test of what fitness means; the Australian Consumer Law’s acceptable quality guarantee similarly leaves “acceptable quality” undefined for a live animal as goods. *Taylor v Lay* shows that courts are already filling this gap case by case. The IHA, framed teleonomically as ten observable functional criteria, is proposed as the breed-independent operational definition both instruments currently lack. The IHA now enables veterinarians to answer regulators’ and courts’ actual question: “Can this animal perform its basic biological functions?”

## 8. Conclusions

Extreme brachycephaly in dogs is the population-level result of sustained artificial selection for traits that matter to humans, at the systematic expense of the biological functions that matter to animals. The VetCompass epidemiological record is unambiguous on the scale of the welfare costs. Dogs from breeds with extreme brachycephaly die years earlier than comparator breeds, carry more simultaneous disorders, and present more frequently to primary care practice with a disease profile dominated by inflammation, congenital pathology, and structural conditions that are present from birth, irresolvable without surgical intervention, and transmitted to every subsequent generation [22,23].

The three frameworks applied in this article converge on a shared account of why. The Five Domains analysis reveals that extreme brachycephaly compromises every welfare domain simultaneously and continuously. Allostatic load theory explains how that simultaneity translates into the documented shorter lifespans: dogs whose bio-regulatory systems are chronically and irreversibly loaded cannot resist the predictable periods of increased regulatory demand that normocephalic dogs generally navigate competently. The teleonome provides the unifying theoretical account, identifying that what is being lost is the integrated biological organisation that was shaped over evolutionary time to detect, evaluate, forecast, and respond to change in adaptive ways. When that organisation is structurally thwarted by the animal’s own morphology, the conditions under which positive welfare becomes possible are themselves undermined.

Although extreme brachycephaly provides a particularly well-studied example, the underlying problem extends beyond any single morphotype. Similar welfare concerns arise, for example, in chondrodystrophic breeds, giant breeds, dogs predisposed to skin-fold disease, and breeds affected by conformational neurological disorders. Furthermore, owners bear escalating veterinary costs and the distress of watching the animals they are attached to struggle with conditions that are predictable at point of purchase. The broader system is self-reinforcing, because consumer demand drives breeding, which entrenches conformational disorder, and the resulting treatment burden becomes part of the economic baseline against which veterinary practice operates. Individual veterinary professionals are often the most committed advocates for change, yet the institutional and financial structures in which they operate have normalised the treatment, rather than the prevention, of conformational disease. These interdependent human, animal, and institutional dynamics place extreme phenotype selection firmly within the scope of a One Welfare account [127].

A corrective pathway was proposed nearly twenty years ago and largely ignored. What has changed is that the legal and regulatory landscape has begun to supply the leverage that professional consensus alone could not generate. Consumer law, animal welfare legislation, and emerging case law are converging to assign veterinarians an enforceable role as arbiters of fitness for purpose. That role requires a standardised, breed-independent, functionally grounded assessment framework. The Innate Health Assessment operationalises the teleonomic principle as ten observable functional criteria. It provides a practical instrument for answering the question that owners, breeders, regulators, and courts must answer. Its logic is transferable to other species. The teleonome reveals wherever artificial selection has compromised the biological functions an animal evolved to perform, from the Pug to the broad-breasted turkey. Selective breeding for extreme phenotypes has not just constrained the teleonome; it has left it in tatters. The frameworks and tools assembled here make that visible, measurable, and resolvable.

Addressing extreme brachycephaly effectively requires coordinated action at several levels. We propose the following concise, point-by-point measures:

- Breeders and kennel/breed clubs: revise breed standards to remove or reword clauses that reward exaggerated skull, muzzle, skin-fold, or body-mass traits, and require pre-breeding functional screening (e.g., using the Innate Health Assessment) rather than conformational judging alone.
- Veterinary regulators and professional bodies: adopt standardised, breed-independent functional assessment tools such as the Innate Health Assessment as the reference standard for fitness-to-breed and pre-purchase advice, and issue clear guidance (as the NSW Veterinary Practitioners Board has begun to do) on recording and disclosing conformational disease findings without minimising them as breed-normal.
- Consumer protection and animal welfare regulators: enforce existing acceptable-quality and fitness provisions at the point of sale, using case law such as Taylor v Lay [2023] NSWCATAP 328 as a template for future enforcement.
- Prospective owners: seek independent, function-based information before purchase, including Innate Health Assessment scores where available, rather than relying on breed reputation or marketing.
- Researchers: extend the epidemiological and Five Domains/allostatic load analysis applied here to extreme phenotypes beyond brachycephaly (e.g., chondrodystrophy, giant breeds, skin-fold and neurological conformations) to support equivalent corrective frameworks.

## Figures and Tables

**Table 1:** Extreme phenotype selection across species: representative examples of the disrupted biological systems and the teleonomic capabilities compromised, with supporting evidence. Breeds listed are those with published epidemiological or morphometric evidence for the stated association; related breeds within the same conformational group (e.g., other Basset-type breeds for the chondrodystrophic row) are likely similarly affected but have not yet been separately studied.

Breed	Species	Extreme Phenotype Selected For	Primary Biological System Disrupted	Teleonomic Capacity Compromised (with Supporting Citations)
Dachshund, Basset Hound, Welsh Corgi (Pembroke and Cardigan)	*Canis lupus familiaris*	Disproportionate vertebral elongation relative to limb length (chondrodystrophy)	Musculoskeletal/locomotory	Sustained movement; FGF4 retrogene drives both artificially selected chondrodystrophic phenotype and premature disc calcification, leading to IVDD (19–24% lifetime prevalence in Dachshunds) [25,26,27]
Show-line German Shepherd Dog	*Canis lupus familiaris*	Extreme hindquarter angulation (sloping topline)	Musculoskeletal/locomotory	Sustained physical work; chronic hip and stifle pathology, degenerative joint disease, early-onset degenerative myelopathy [20,28]
Giant breeds (English Mastiff, Great Dane, Dogue de Bordeaux, Irish Wolfhound)	*Canis lupus familiaris*	Extreme body mass	Cardiovascular/musculoskeletal	Sustainable cardiac output and longevity; dilated cardiomyopathy, osteosarcoma, gastric dilatation-volvulus; median longevity 5.5–6.0 years [28,29,30,31]
Cavalier King Charles Spaniel	*Canis lupus familiaris*	Foreshortened skull relative to normally sized brain and spinal cord	Neurological	Pain-free neurological function; Chiari-like malformation and syringomyelia (219× odds vs. dogs overall); chronic, often unresolvable neuropathic pain [32,33,34]
Shar-Pei, Chow Chow, Neapolitan Mastiff, Clumber Spaniel, Saint Bernard, English Bulldog	*Canis lupus familiaris*	Ectropion- and entropion-predisposing eyelid conformation	Ocular	Ocular surface integrity; chronic discomfort, corneal ulceration, risk of vision loss; entropion up to 15.4% annual prevalence (Shar-Pei) [35,36,37,38]
Shar-Pei, Neapolitan Mastiff, Bloodhound, English Bulldog, French Bulldog	*Canis lupus familiaris*	Extreme skin folding	Integumentary	Barrier function and inflammatory resolution; chronic dermatitis and pruritus with no resolution pathway (English Bulldog: >6% annual skin-fold dermatitis vs. 0.2% in crossbreds) [20,39,40]
Myotonic (Tennessee fainting) goat	*Capra aegagrus hircus*	CLCN1 missense mutation causing myotonia congenita	Neuromuscular	Flight response; tetanic muscle contraction and collapse on startle [41]
Broad-breasted commercial turkey	*Meleagris gallopavo domesticus*	Pectoralis muscle mass 2.8× that of wild birds relative to body mass	Musculoskeletal/reproductive	Natural mating; physically impossible, requiring exclusive reliance on artificial insemination [42,43]
Commercial broiler chicken	*Gallus gallus domesticus*	>300% increase in growth rate over fifty years	Musculoskeletal	Weight-bearing locomotion; tibial dyschondroplasia, femoral head necrosis, spontaneous fracture; broodstock require permanent nutritional restriction to survive to breeding age [44,45]
Roller and tumbler pigeon (parlour tumbler strains)	*Columba forma domestica*	Selection for in-flight somersaulting	Neurological/locomotory	Normal flight; compulsive tumbling on attempted take-off, with disorientation and injury risk [46]

## Data Availability

No new data were created or analysed in this study. This article synthesises data and findings already reported in the cited publications.

## Abbreviations

The following abbreviations are used in this manuscript:	
BOAS	Brachycephalic obstructive airway syndrome
HPA	Hypothalamic–pituitary–adrenal
URT	Upper respiratory tract
GI	Gastrointestinal
CSF	Cerebrospinal fluid
DEFR+U	Detection, evaluation, forecasting, response + updating priors
AU	Allostatic units
D1–D5	Domains 1–5 of the Five Domains Model
The following terms recur throughout this article and are collected here for ease of reference; each is also introduced and explained in the context of its first use in the main text.	
**Affectome**	The subset of an animal’s teleonomic traits responsible for generating, qualifying, and prioritising its affective (emotional) responses; the totality of an animal’s capacity for affective experience.
**Affordances**	Opportunities for action that arise from the relationship between an animal’s environment and its own physical and behavioural capabilities (e.g., a structurally normal airway affords effective panting).
**Allostasis**	The physiological and behavioural adjustments through which animals respond to predictable and unpredictable stressors aimed at maintaining stability.
**Allostatic load**	The cumulative biological cost incurred through repeated or sustained allostatic responses; when recovery from this cost is impaired, it can progress to allostatic overload.
**Brachycephalic**	Having a foreshortened facial skeleton (skull) relative to a normally proportioned (mesocephalic) skull, such that the muzzle is markedly reduced relative to cranial length.
**Cephalic index**	A measure of skull shape, calculated from skull width relative to skull length, used to classify dogs along the brachycephalic-mesocephalic-dolichocephalic continuum.
**DEFR+U cycle**	A conceptualisation of teleonomic regulatory processes as a repeating cycle of detection, evaluation, forecasting, and response, followed by updating of priors.
**Dolichocephalic**	Having an elongated facial skeleton (skull) relative to a normally proportioned (mesocephalic) skull (e.g., sighthound breeds).
**Mesocephalic**	Having a normally proportioned facial skeleton (skull), intermediate between the brachycephalic and dolichocephalic extremes.
**Teleonome**	The integrated, evolved regulatory architecture that enables an animal to maintain viability and pursue its biological goals (telos) through physiological, behavioural, and adaptive bio-regulatory processes.
**Teleonomic competence (capacity)**	The functional ability of an animal’s teleonome to resolve or avoid regulatory challenges effectively, i.e., whether ongoing experience is building or eroding this capacity.
